# Supplementary material for: The global loss of floristic uniqueness
Source: Nat Commun. 2021 Dec 15;12:7290. doi: 10.1038/s41467-021-27603-y (PMC8674287; doi:10.1038/s41467-021-27603-y)
Supplement: Supplementary file 1 — Supplementary information [file 41467_2021_27603_MOESM1_ESM.pdf]

Supplementary information for

The global loss of floristic uniqueness

Qiang Yang*, Patrick Weigelt, Trevor S. Fristoe, Zhijie Zhang, Holger Kreft, Anke Stein, Hanno Seebens, Wayne Dawson, Franz Essl, Christian König, Bernd Lenzner, Jan Pergl, Robin Pouteau, Petr Pyšek, Marten Winter, Aleksandr L. Ebel, Nicol Fuentes, Eduardo L. H. Giehl, John Kartesz, Pavel Krestov, Toomas Kukk, Misako Nishino, Kupriyanov Andrey Nikolaevich, Jose Luis Villaseñor, Jan J. Wieringa, Abida Zeddam, Elena Zykova & Mark van Kleunen

**Email of the corresponding author: qiang.yang@uni-konstanz.de*

Supplementary Methods: Contributions of the most widespread alien plants to floristic homogenization

To test whether floristic homogenization is driven mainly by the most widely naturalized species, we compared the observed homogenization extent to those calculated after removal of the most widely naturalized species and after removal of the least widely naturalized ones. We first ranked species within the global naturalized species pool by the number of regions in which they are naturalized. We then conducted two “experiments” of species removal. In the first, we removed the 10% most widely naturalized species from all regions, and, in the second, we removed the 10% least widely naturalized species. We then calculated the homogenization in pairwise regions and compared them to that calculated without removal of naturalized species.

Notes for the display of P value in supplementary tables and supplementary figures: The *P* value smaller than 2.2×10^{-16} (the minimum *P* value that returned from the function *summary* in R) is displayed as 0.

Supplementary Table 1 | Single-predictor log-binomial GLMs testing how taxonomic and phylogenetic similarities depend on geographic distance and climatic distance. Columns from left to right: the response variable of the GLM (i.e. pairwise similarity between regions), the predictor (geographic distance or climatic distance), the formula of the regression model (intercepts fixed at zero are indicated with + 0), the model coefficient estimate, the standard error of the coefficient estimate, and the *P* value indicating the significance of the predictor.

**Response**	**Predictor**	**Formula**	**Coefficient Estimate**	**Standard Error**	***P* value**
$SimTax_{native}$	geographic distance	y ~ x + 0	-3.87×10^{-4}	1.43×10^{-6}	0
$SimTax_{native+naturalized}$	geographic distance	y ~ x + 0	-2.58×10^{-4}	8.04×10^{-7}	0
$SimPhyl_{native}$	geographic distance	y ~ x + 0	-9.89×10^{-5}	2.99×10^{-7}	0
$SimPhyl_{native+naturalized}$	geographic distance	y ~ x + 0	-9.26×10^{-5}	2.83×10^{-7}	0
$SimTax_{native}$	climatic distance	y ~ x	-4.46×10^{-1}	4.34×10^{-3}	0
$SimTax_{native+naturalized}$	climatic distance	y ~ x	-3.38×10^{-1}	3.12×10^{-3}	0
$SimPhyl_{native}$	climatic distance	y ~ x	-7.67×10^{-2}	1.34×10^{-3}	0
$SimPhyl_{native+naturalized}$	climatic distance	y ~ x	-8.67×10^{-2}	1.28×10^{-3}	0

Supplementary Table 2 | MRM models analysing how taxonomic homogenization (Htax) and phylogenetic homogenization (Hphyl) relate to geographic distance (Geo_dist), climatic distance (clim_dist) and administrative relations (admin). Columns from left to right: the response of the MRM (i.e. pairwise homogenization between regions), the predictor, the model coefficient estimate, the standard error of the coefficient estimate, and the *P*-value indicating the significance of the predictor.

Response	Predictor	Coefficient Estimate	Standard Error	*P*-value
Htax	Intercept	1.115	0.003	0
	Geo_dist	0.467	0.003	0
	Clim_dist	-0.049	0.003	0
	Admin_dependency	0.247	0.006	0
	Admin_same country	1.701	0.050	0
	Geo_dist:Clim_dist	-0.086	0.003	0
	Geo_dist:Admin_dependency	0.166	0.006	0
	Geo_dist:Admin_same country	1.140	0.035	0
	Clim_dist:Admin_dependency	0.077	0.007	0
	Clim_dist:Admin_same country	0.917	0.058	0
	Geo_dist:Clim_dist: Admin_dependency	0	0.006	0.963
	Geo_dist:Clim_dist: Admin_same country	0.615	0.035	0
Hphyl	Intercept	0.081	0	0
	Geo_dist	0.037	0	0
	Clim_dist	-0.039	0	0
	Admin_dependency	-0.009	0.001	0
	Admin_same country	-0.080	0.006	0
	Geo_dist:Clim_dist	-0.018	0	0
	Geo_dist:Admin_dependency	0.014	0.001	0
	Geo_dist:Admin_same country	-0.049	0.004	0
	Clim_dist:Admin_dependency	0.001	0.001	0.502
	Clim_dist:Admin_same country	-0.017	0.007	0.018
	Geo_dist:Clim_dist: Admin_dependency	-0.003	0.001	2.28×10^{-4}
	Geo_dist:Clim_dist: Admin_same country	-0.018	0.004	3.53×10^{-5}

Supplementary Table 3 | The 19 bioclimatic variables extracted for the quantification of climatic distance. Columns from left to right: the name of the bioclimatic variable, the transformation function that made the variable best approximate a normal distribution, the D value of the Kolmogorov-Smirnov test for the best transformation, and the distribution of the transformed data.

Bioclimatic Variable	Best Transformation Function	D Value of Kolmogorov-Smirnov Test	Distribution of Transformed Data
Annual Mean Temperature	inverse log	0.086	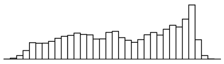
Annual Precipitation	cube root	0.026	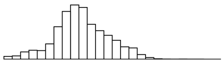
Isothermality	boxcox	0.066	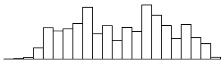
Max Temperature of Warmest Month	boxcox	0.062	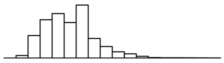
Mean Diurnal Range	no transformation	0.034	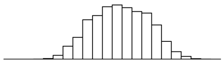
Mean Temperature of Coldest Quarter	inverse log	0.080	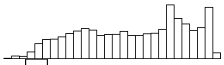
Mean Temperature of Driest Quarter	inverse log	0.081	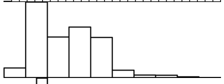
Mean Temperature of Warmest Quarter	inverse log	0.055	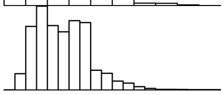
Mean Temperature of Wettest Quarter	inverse log	0.063	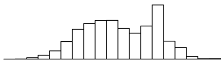
Min Temperature of Coldest Month	inverse log	0.071	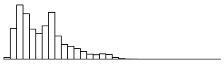
Precipitation of Coldest Quarter	boxcox	0.027	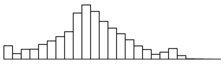
Precipitation of Driest Month	boxcox	0.073	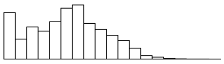
Precipitation of Driest Quarter	boxcox	0.037	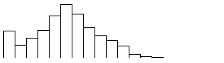
Precipitation of Warmest Quarter	square root	0.037	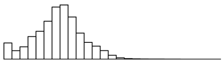
Precipitation of Wettest Month	boxcox	0.043	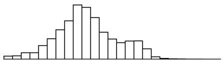
Precipitation of Wettest Quarter	boxcox	0.040	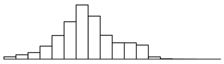
Precipitation Seasonality	square root	0.022	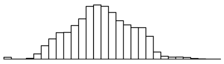
Temperature Annual Range	square root	0.036	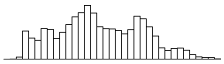
Temperature Seasonality	square root	0.043	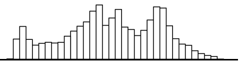

Supplementary Table 4 | Databases and datasets used in this study. Columns from left to right represent the name, the version, the latest access date and the approach of access for each database or dataset.

Name	Version	Latest Date of Access (DD-MM-YYYY)	Approach of Access
GloNAF	v1.2	23-09-2020	via R package 'RMySQL' (version 0.10.20)
GIFT	v2.0	23-09-2020	via R package 'RMySQL' (version 0.10.20)
ALLMB	v0.1	18-01-2019	downloaded from https://github.com/FePhyFoFum/big_seed_plant_trees/releases
The Plant List	v1.1	17-02-2020	downloaded lists of flowering plants from http://www.theplantlist.org/1.1/browse/A/
TRADHIST	no version number	01-07-2019	downloaded from http://www.cepii.fr/CEPII/en/bdd_modele/presentation.asp?id=32
Worldclim	v2	14-03-2020	via R package 'raster' (version 3.0-7). The database could also be downloaded from http://www.worldclim.com/version2

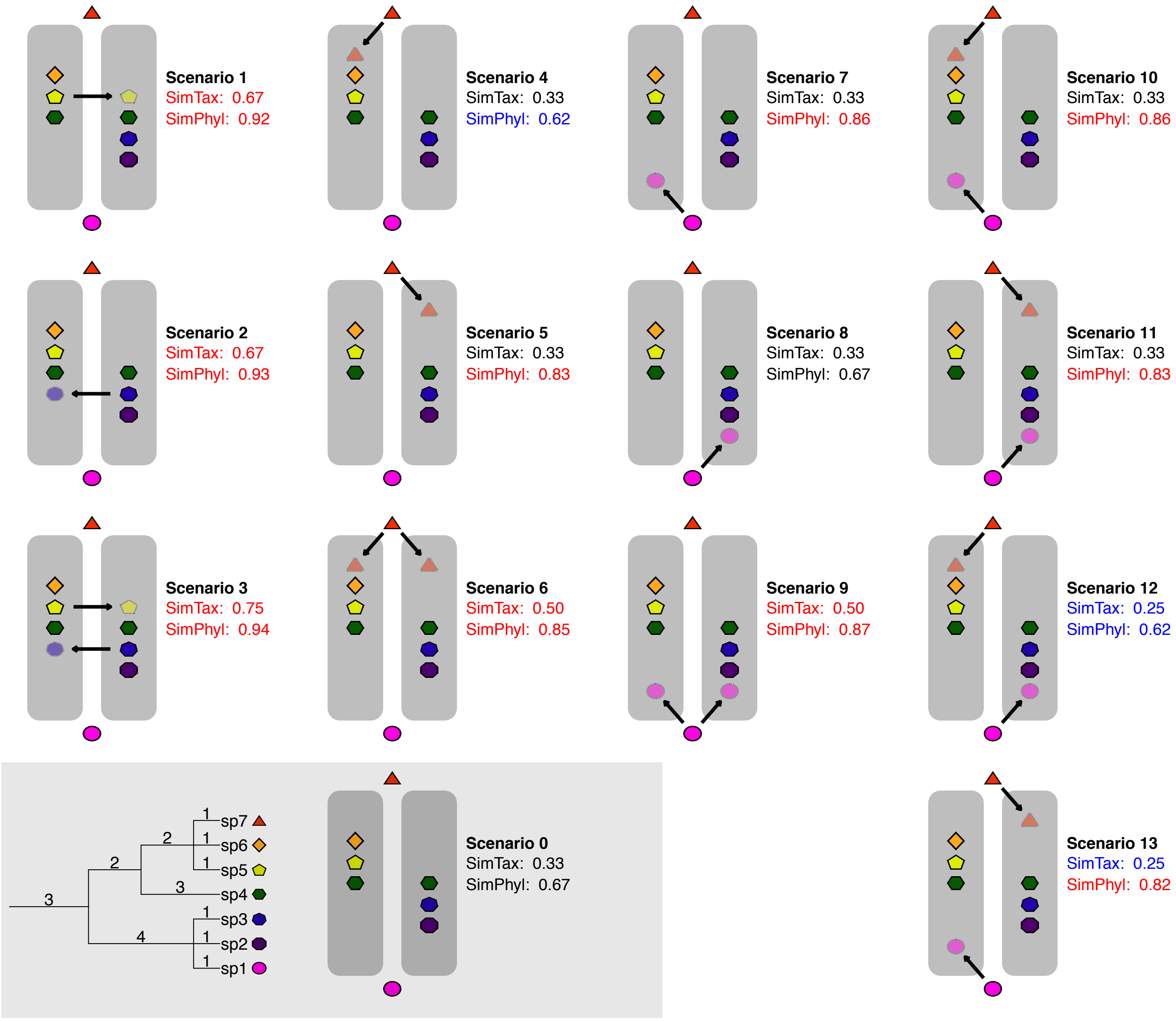

Supplementary Fig. 1 | Illustration of 13 different scenarios of species naturalization and their consequences for taxonomic and phylogenetic similarities between regions. In each scenario, the triangle and circle represent species that are alien to both regions, while other symbols represent native species. The phylogenetic relatedness among the example species is shown in the light gray box at the bottom left corner. Scenarios 1-3 show examples of reciprocal exchange of species between the two regions, i.e. where a species native to one region naturalizes in the other. Scenarios 4-9 show examples where one species alien to both regions naturalizes in only one of the two regions (4-5, 7-8) or in both regions (6, 9). Scenarios 10-13 show examples where two species alien to both regions naturalize both in the same region (10-11) or in different regions (12-13). The taxonomic similarity (*SimTax*) and phylogenetic similarity (*SimPhyl*) between the regions in each scenario were calculated. Red font indicates that the similarity is higher than that in scenario 0, in which there is no naturalization, and a blue font indicates that it is lower.

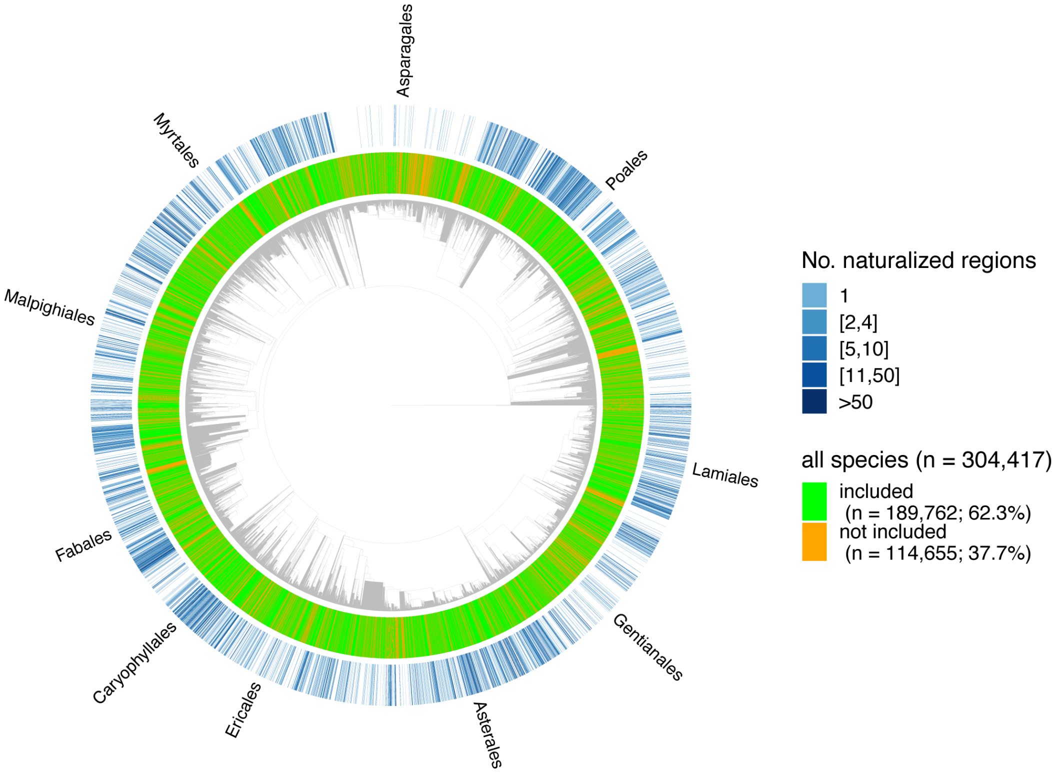

Supplementary Fig. 2 | The phylogeny of all accepted angiosperm species in The Plant List. In the inner ring, green bars indicate the positions of species that are included in our dataset, while orange bars indicate positions of the species that are not included in the analysis. The latter most likely occur in regions for which we had no complete native and alien species lists (see Fig. 4). In the outer ring, species that are naturalized in other regions are marked by blue bars, with the intensity of the blue tone indicating the number of regions in which the species is naturalized.

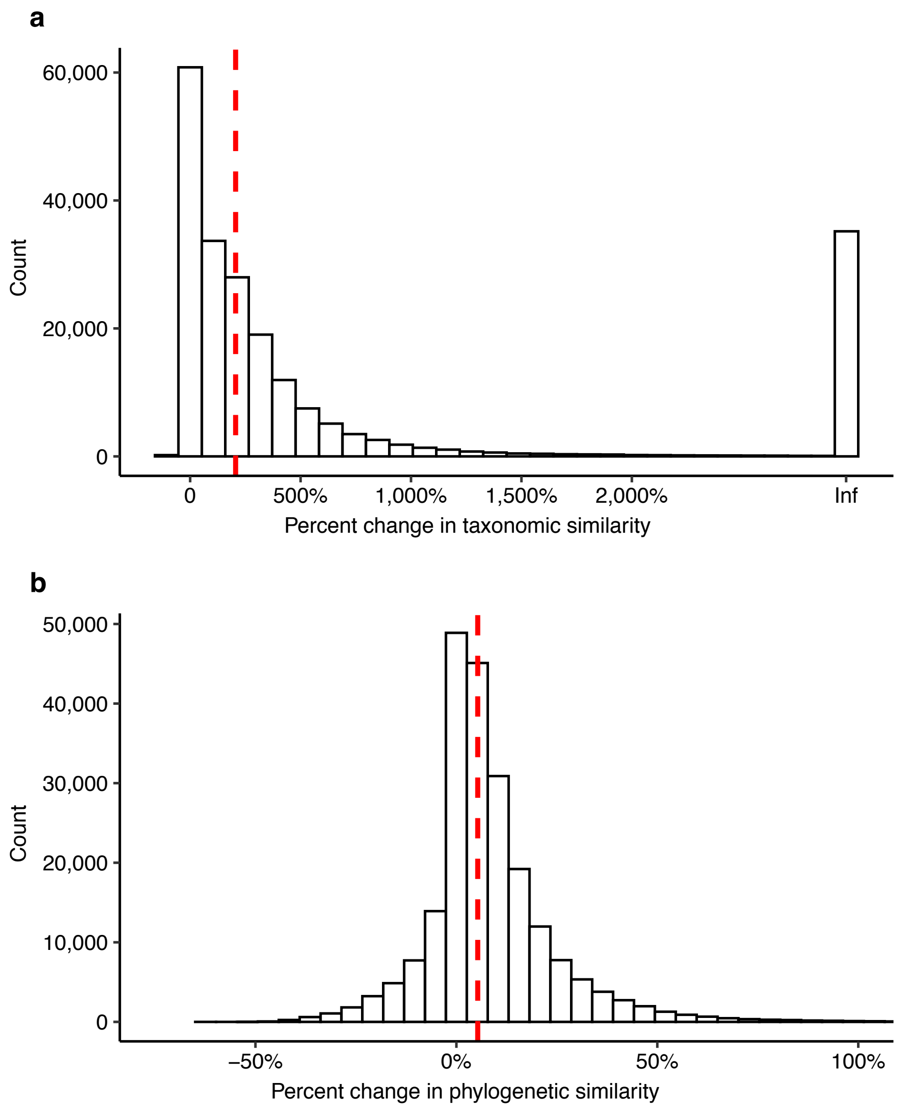

Supplementary Fig. 3 | Histograms displaying the distribution of the change (%) in pairwise taxonomic similarity (a) and pairwise phylogenetic similarity (b) between regions caused by naturalized alien species. Changes in taxonomic and phylogenetic similarity were calculated as $((SimTax_{native+naturalized} - SimTax_{native}) / SimTax_{native} * 100\%)$ and $((SimPhyl_{native+naturalized} - SimPhyl_{native}) / SimPhyl_{native} * 100\%)$, respectively. The dashed red lines indicate median values. The Infinite value (Inf) in **(a)** corresponds to the situation when $SimTax_{native}$ is zero. Results of the one-sided Wilcoxon signed-rank test showed that both changes in taxonomic and phylogenetic similarity are significantly larger than zero (taxonomic: $n = 216{,}153$, $V = 2.31\times10^{10}$, $P = 0$; phylogenetic: $n = 216{,}153$, $V = 1.88\times10^{10}$, $P = 0$).

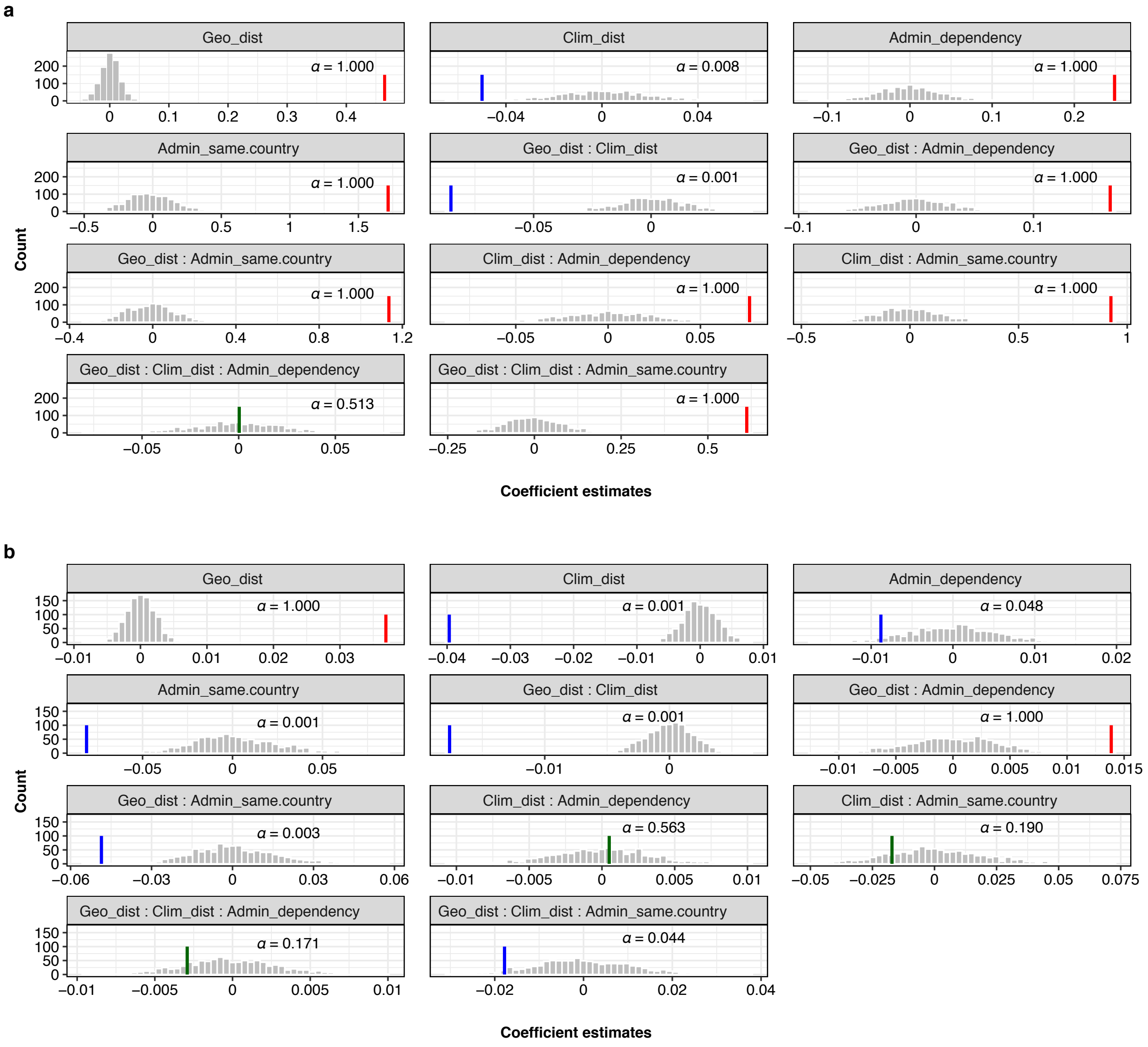

Supplementary Fig. 4 | The null distribution (*n* = 999) of the coefficient estimates for the MRM model analysing how taxonomic (a) and phylogenetic (b) homogenization relate to geographic distance, climatic distance (centroid approach) and the administrative relationship between regions. The null distribution was produced by simultaneously shuffling the rows and columns of the response matrix**.** The percentile of the coefficient estimate using original data (α) in the null distribution is given. The red vertical lines represent significant positive coefficient estimates (α > 0.95), the blue vertical lines represent significant negative coefficient estimates (α < 0.05), and the green vertical lines represent non-significant estimates (0.05 <= α <= 0.95).

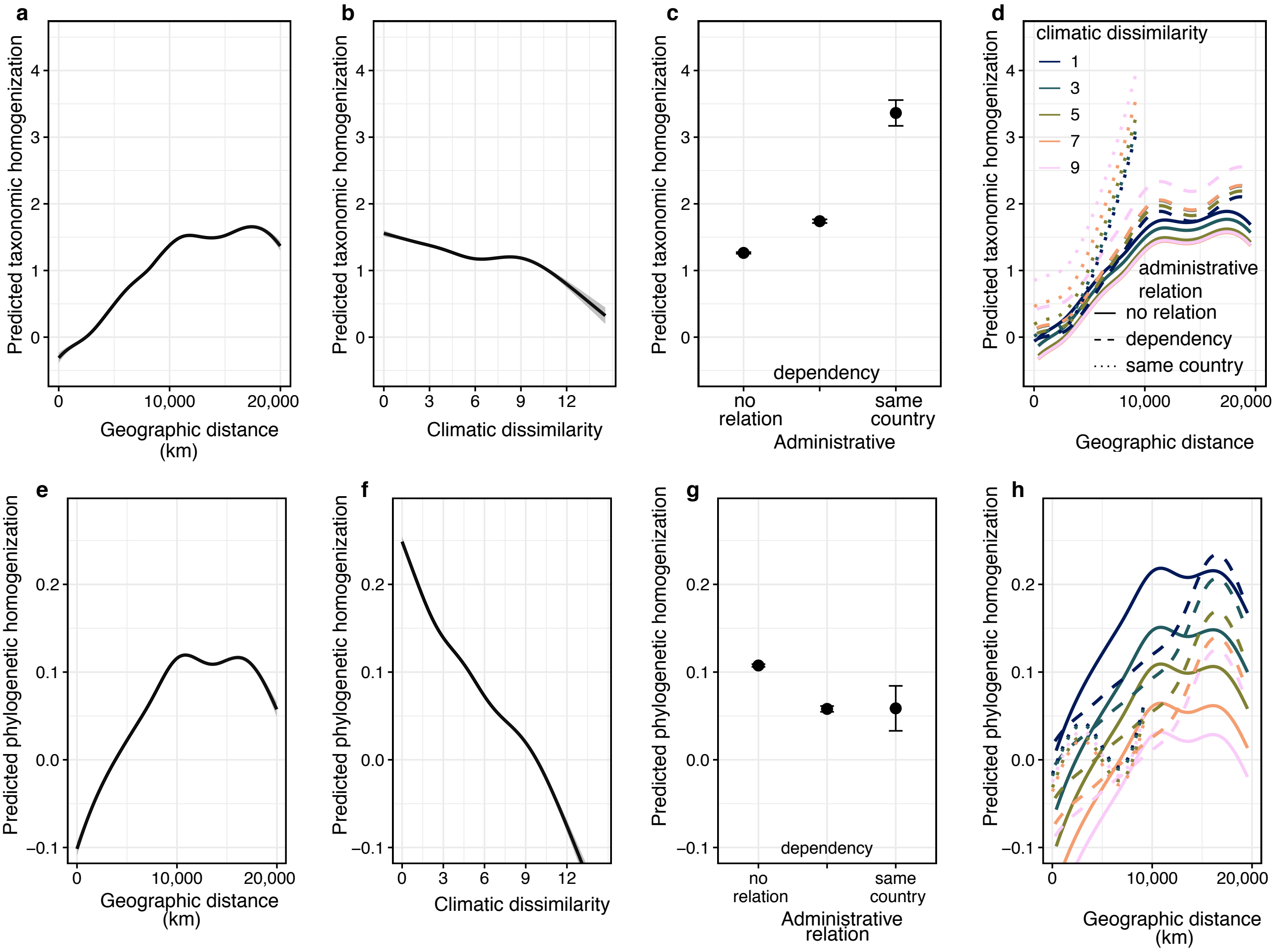

Supplementary Fig. 5 | GAM results on the association of the degree of taxonomic and phylogenetic homogenization between regions with their geographical distance, climatic distance and the administrative relationship (*n* = 216,153). **a-c** and **e-g**, Plots display the main effects of geographical distance, climatic distance and administrative relationships on the degree of taxonomic and phylogenetic homogenization. **d** and **h**, Plot shows possible interactive effect between the three explanatory variables. The error bands in **a-b** and **e**-f and the error bars in **c** and **g** represent the 95% confidence interval of the predicted homogenization value. For the GAM, smooth functions were applied to all continuous explanatory terms and their interactions, but not to the main effect of the categorical variable 'administrative relationship'. We tested the robustness of the results using different values of the smoothing term (set to 5, 6 and 7), and the results were qualitatively consistent. We, therefore, set the smoothing term to 6 for the final model. The results were largely consistent with those of the MRM models (Fig. 3c,f). However, while the MRM model revealed a negative effect of climatic distance on taxonomic homogenization for regions with an administrative dependency (at least for geographically distant regions), the GAM showed the opposite. In addition, while the MRM revealed a negative effect of climatic distance on phylogenetic homogenization for regions belonging to the same country, the GAM revealed the opposite. We see three possible reasons for these discrepancies: (1) The relationship between homogenization and climatic distance is not linear (as assumed by the MRM models). (2) GAMs, in contrast to the MRM models, do not account for non-independence of the data points. (3) Some of the environmental distances are relatively rare for regions that are part of the same country or have a dependency relationship (see Supplementary Fig. 6), and that as a consequence homogenization is predicted for climatic similarities that do not exist or are rare.

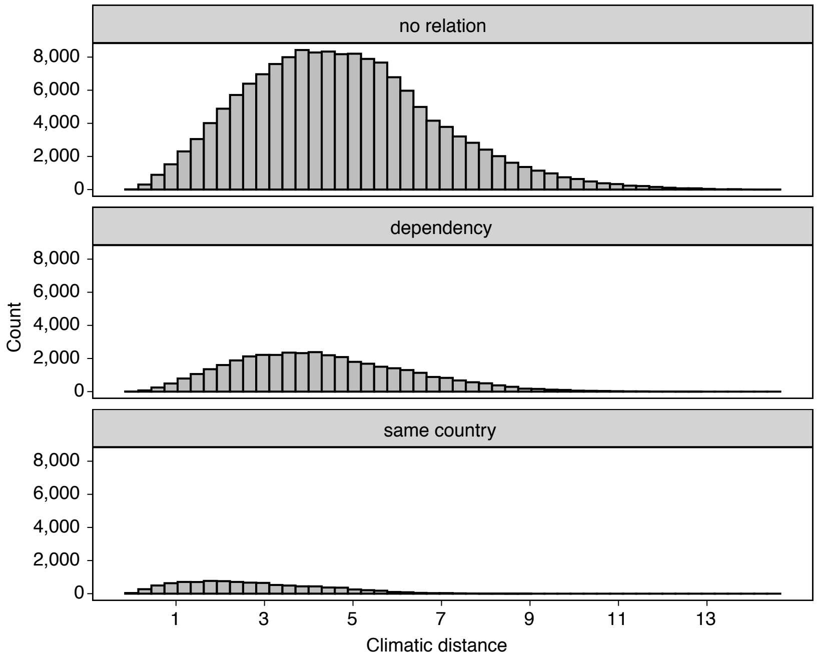

Supplementary Fig. 6 | Histograms displaying the distribution of climatic distances between region pairs of different administrative relationships.
Panels from the bottom: pairs of regions 1) belonging to the same country (*n* = 10,362), 2) that have a current or past dependency on the other (*n* = 41,916), and 3) without administrative ties (*n* = 163,875).

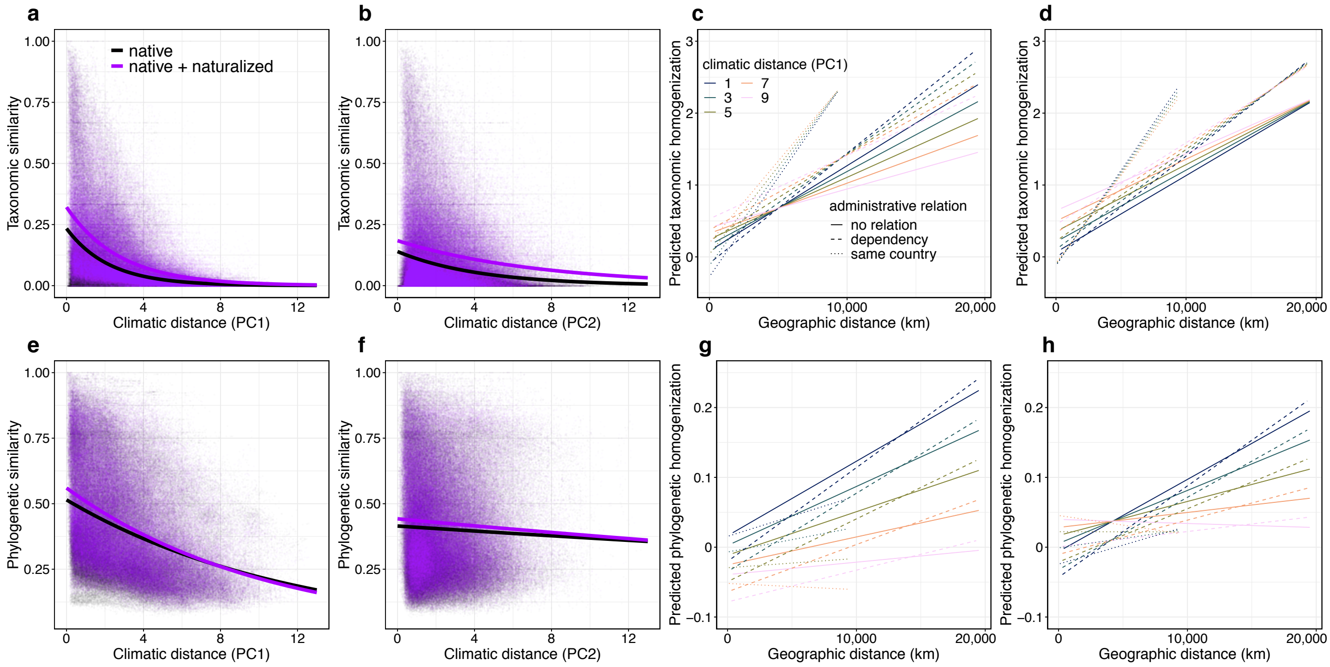

Supplementary Fig. 7 | Taxonomic and phylogenetic homogenization in relation to climatic distance (PC1 and PC2). PC1 is mainly related to temperature variables and PC2 is mainly related to precipitation variables (see Supplementary Fig. 14). Taxonomic (**a, b**) and phylogenetic (**e, f**) similarities of native species ($SimTax_{native}$ and $SimPhyl_{native}$) (black) and of natives and naturalized species combined ($SimTax_{native+naturalized}$ and $SimPhyl_{native+naturalized}$) (purple) versus climatic distance between the two regions in a pair (*n* = 216,153 for each type of similarity). The curves in **a**, **b**, **e** and **f** are from the fitted GLMs (see Methods). **c**, **d**, **g** and **h** , The degrees of taxonomic and phylogenetic homogenization as predicted by the multiple regression on distance matrices showing the effects of geographic distance, climatic distance (PC1 and PC2), the administrative relationship and their interactions. To better visualize the predicted response of homogenization to the predictors, we arbitrarily set climatic distance values to 1, 3, 5, 7, 9.

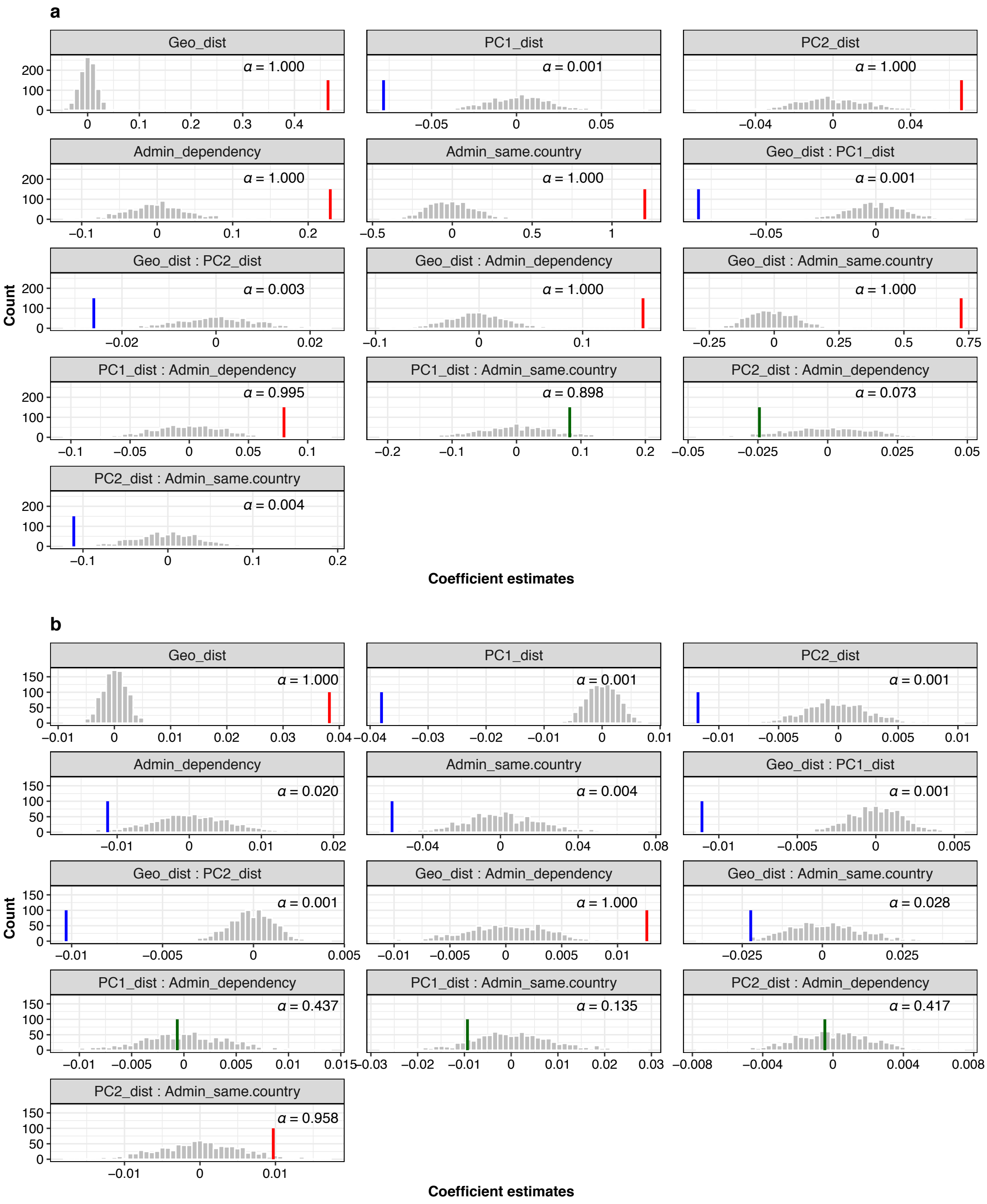

Supplementary Fig. 8 | The null distribution (*n* = 999) of the coefficient estimates for the MRM model analysing how taxonomic (a) and phylogenetic (b) homogenization relate to geographic distance, climatic distance (PC1 and PC2) and the administrative relationship between regions. The null distribution was produced by simultaneously shuffling the rows and columns of the response matrix**.** The percentile of the coefficient estimate using original data (α) in the null distribution is given. The red vertical lines represent significant positive coefficient estimates (α > 0.95), the blue vertical lines represent significant negative coefficient estimates (α < 0.05), and the green vertical lines represent non-significant estimates (0.05 <= α <= 0.95).

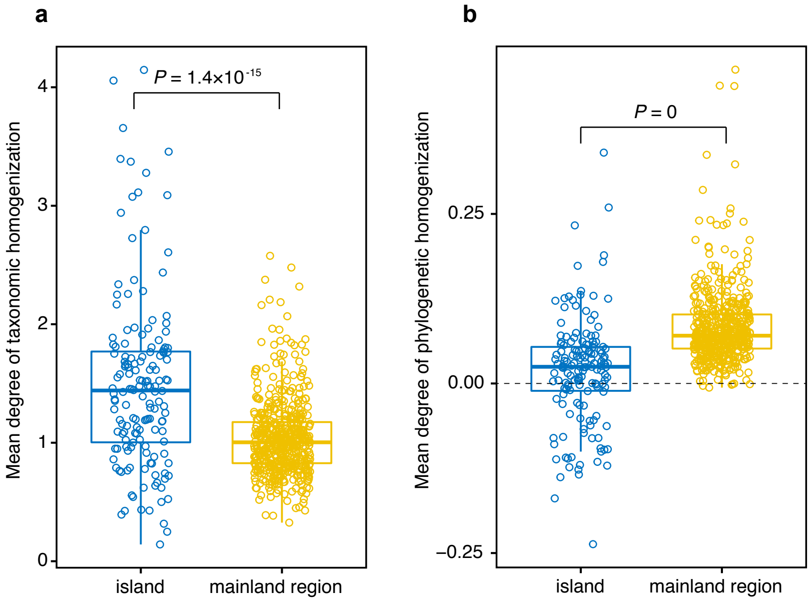

Supplementary Fig. 9 | Boxplots comparing the mainland regions (*n* = 503) and islands (*n* = 154) in their mean degrees of taxonomic (a) and phylogenetic (b) homogenization. One region, Chile, includes both mainland area and island, and it is therefore not included in this figure. In each boxplot, the box indicates the interquartile range (IQR), the line within the box indicates the median and the whiskers indicate the range of the observed values between the interval (Q1 - 1.5 * IQR, Q3 + 1.5 * IQR), where Q1 and Q3 are the first and the third quantile, respectively. The *P* value of the comparison using the one-sided Mann-Whitney-Wilcoxon test is displayed on the graph.

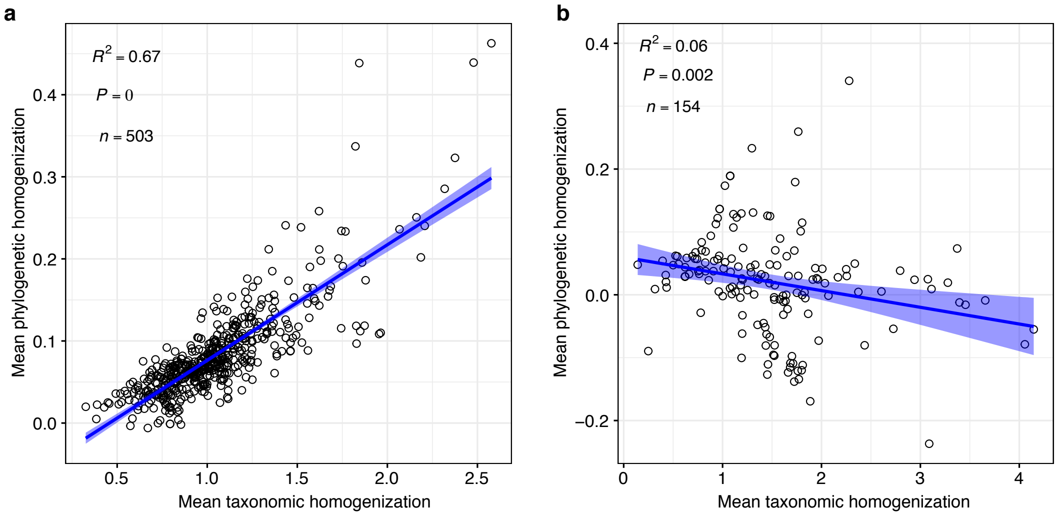

Supplementary Fig. 10 | The relationship between mean taxonomic homogenization and mean phylogenetic homogenization for mainland regions (a) and islands (b). For each of the regions (n = 658), we calculated its average taxonomic and phylogenetic homogenization with other regions as a consequence of alien species naturalization. Points in both **a** and **b** represent different regions, and the line displays the fitted line from a linear regression analysis. The R^2, P-value and the sample size n of the regression are given. One region (Chile) includes both mainland area and islands, and is not included in the figure.

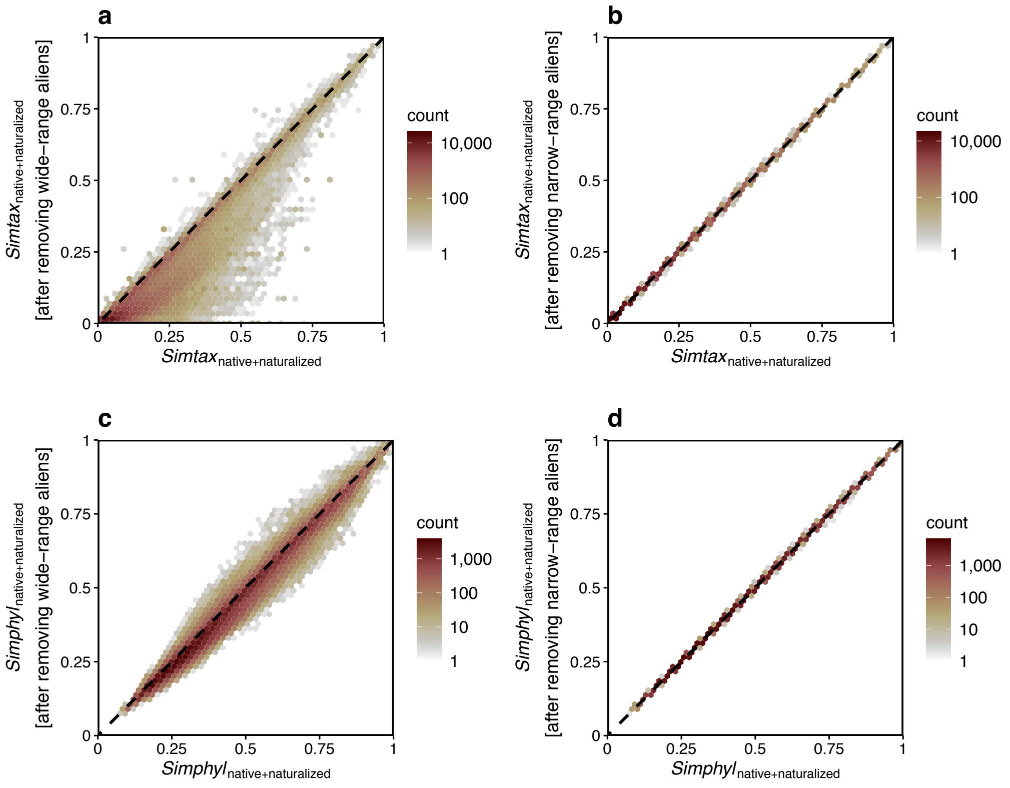

Supplementary Fig. 11 | Hexagonal bin plots comparing the taxonomic similarity (a, b) and phylogenetic similarity (c, d) between region pairs calculated using both the native and the full naturalized flora to that calculated using both the native and the naturalized flora after removing the 10% most widely naturalized species (a, c) and to that after removing the 10% least widely naturalized species (b, d). The dashed line is the identity line. The data (n = 216,153 region × region comparisons) are binned into hexagonal cells to improve figure readability. Removal of the 10% most widely naturalized species resulted in a significant lower floristic similarity (*SimTax*: n = 216,153, $V = 2.10 \times 10^{10}$, P = 0; *SimPhyl*: n = 216,153, $V = 2.08 \times 10^{10}$, P = 0). Removal of the 10% least widely naturalized species resulted in a slightly higher, but significant, floristic similarity (*SimTax*: n = 216,153, $V = 3.21 \times 10^{8}$, P = 0; *SimPhyl*: n = 216,153, $V = 1.32 \times 10^{9}$, P = 0). The statistical comparisons were made using one-sided Wilcoxon signed rank test for paired data. For more details about the removal of the naturalized species, see Supplementary Methods.

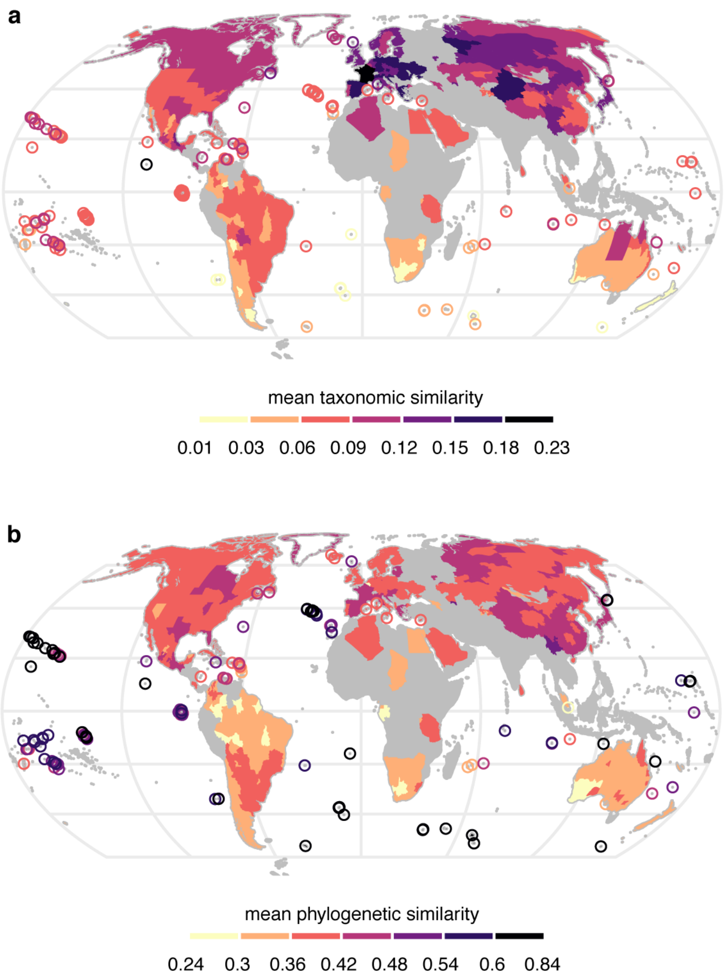

Supplementary Fig. 12 | Maps showing the mean similarity of the native flora of each region to the native flora of all other regions. For each of the regions (*n* = 658), we calculated the mean similarity of its native flora to those of other regions. The color gradient represents the degree of mean taxonomic similarity (**a**) and mean phylogenetic similarity (**b**). Islands smaller than 10,000 km^2 (*n* = 137) were labelled with circles to ensure their visibility on the map.

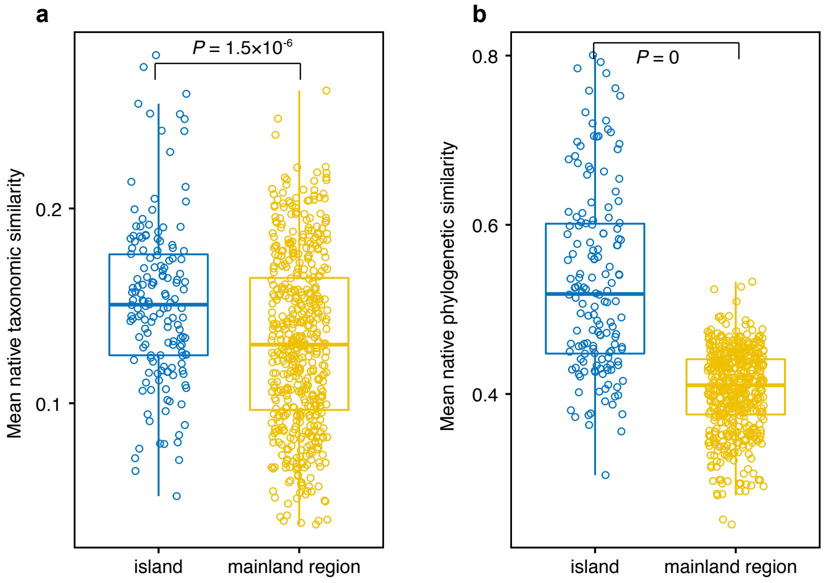

Supplementary Fig. 13 | Boxplots comparing the mean taxonomic (a) and phylogenetic (b) similarities of the native floras of mainland regions (n = 503) and of islands (n = 154) to other regions. One region, Chile, includes both mainland area and islands, and it is therefore not included in this figure. In each boxplot, the box indicates the interquartile range (IQR), the line within the box indicates the median and the whiskers indicate the range of the observed values between the interval (Q1 - 1.5 * IQR, Q3 + 1.5 * IQR), where Q1 and Q3 are the first and the third quantile, respectively. The P value of the comparison using the one-sided Mann-Whitney-Wilcoxon test is displayed on the graph.

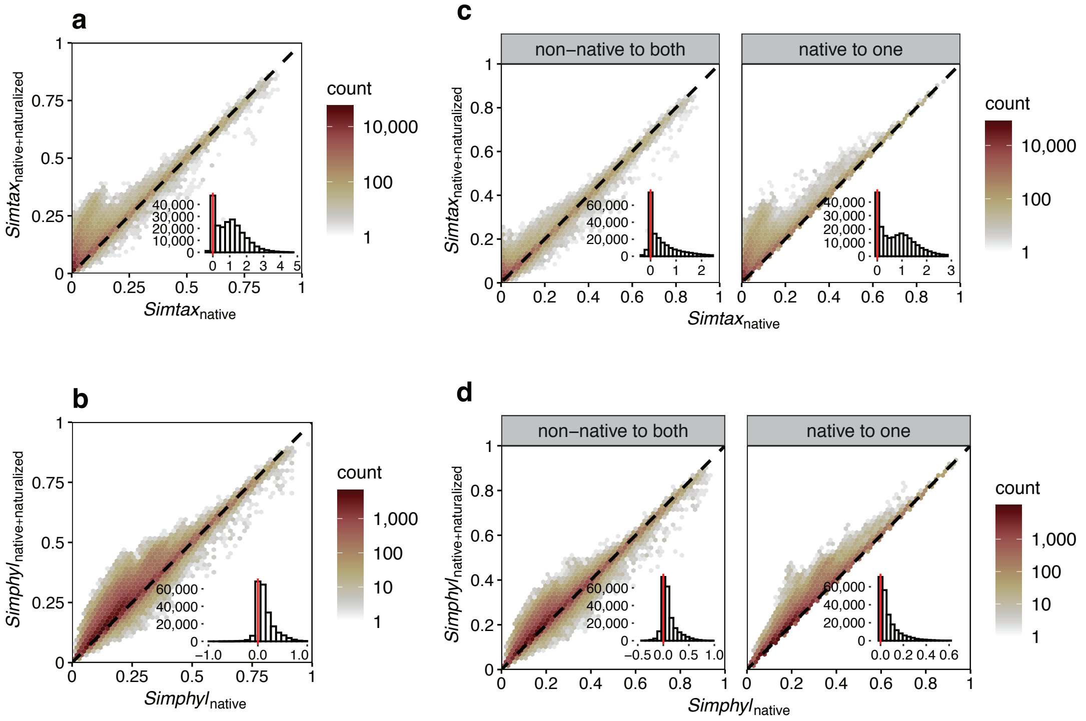

Supplementary Fig. 14 | The degree of taxonomic (a,c) and phylogenetic (b,d) homogenization of regional floras that is quantified using the "Sørensen" similarity index. a and **b,** hexagonal bin plots showing the change in taxonomic and phylogenetic similarities, respectively, driven by all naturalized alien species. The dashed line indicates where the similarity of two native floras (Sim_{native}) equals the similarity of their native and naturalized floras combined ($Sim_{native+naturalized}$). The data (n = 216,153 region × region comparisons) are binned into hexagonal cells to improve figure readability. The inset histograms show the frequency distributions of the degree of taxonomic and phylogenetic homogenization, respectively. **c** and **d** also show the degree of taxonomic and phylogenetic homogenization, respectively, but for the subsets of naturalized species that are either restricted to species that are alien to both regions ('alien to both') or to species that are native to only one of the two regions ('native to one').

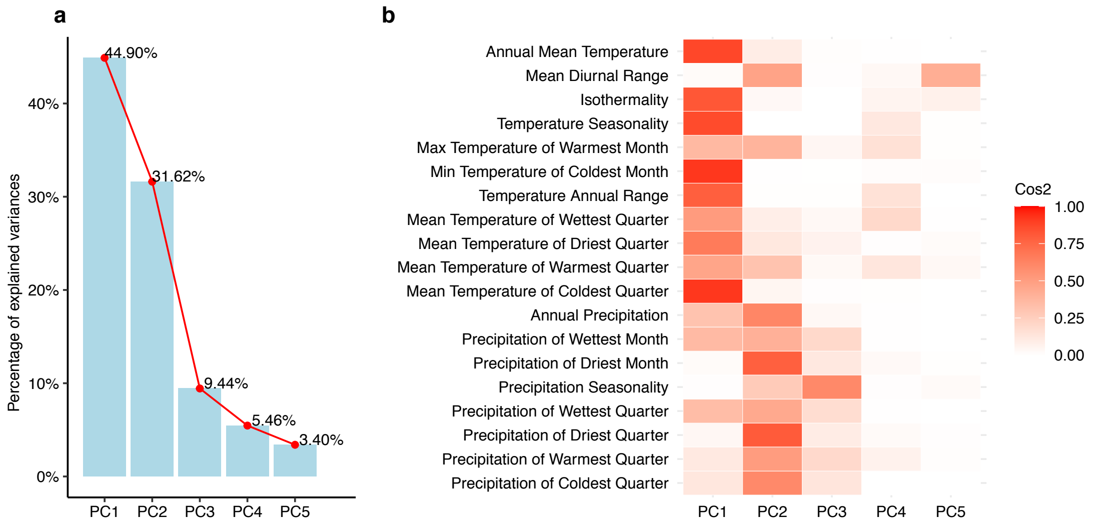

Supplementary Fig. 15 | Results of principal component analysis (PCA) on the 19 bioclimatic variables. a, A bar plot displaying the percentages of variance in the 19 bioclimatic variables explained by the first five principal components (PC1-PC5). **b,** A heatmap showing the correlation between the first five principal components and the bioclimatic variables, with a high Cos2 indicating a good representation of the bioclimatic variable on the principal component.

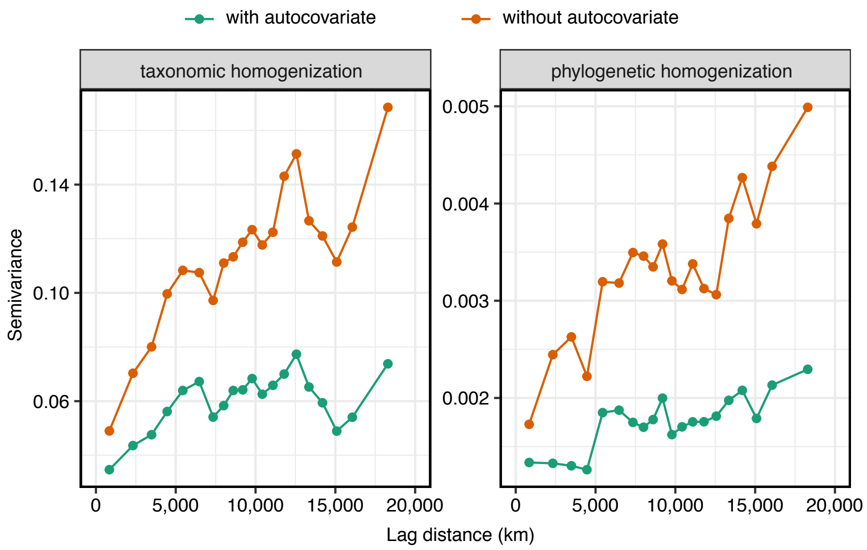

Supplementary Fig. 16 | Semivariogram for detecting spatial autocorrelation in the residuals of the model analysing how the mean degree of homogenization relates to native species richness, naturalized species richness, the proportion of endemic species, the donor score, the size of the region, and whether the region is an island or a mainland region (*n* = 657). In the models without autocovariate (orange lines), strong spatial autocorrelations in the model residuals were detected. In the models including an autocovariate (green lines), spatial autocorrelations in the model residuals were largely reduced.
