# Supplementary material for: The global loss of floristic uniqueness
Source: Nat Commun. 2021 Dec 15;12:7290. doi: 10.1038/s41467-021-27603-y (PMC8674287; doi:10.1038/s41467-021-27603-y)
Supplement: Supplementary file 3 — Reporting Summary [file 41467_2021_27603_MOESM3_ESM.pdf]

nature research

Corresponding author(s): Qiang Yang

Last updated by author(s): Nov 14, 2021

Reporting Summary

Nature Research wishes to improve the reproducibility of the work that we publish. This form provides structure for consistency and transparency in reporting. For further information on Nature Research policies, see our Editorial Policies and the Editorial Policy Checklist.

Statistics

For all statistical analyses, confirm that the following items are present in the figure legend, table legend, main text, or Methods section.

n/a	Confirmed	
☐	☒	The exact sample size (*n*) for each experimental group/condition, given as a discrete number and unit of measurement
☒	☐	A statement on whether measurements were taken from distinct samples or whether the same sample was measured repeatedly
☐	☒	The statistical test(s) used AND whether they are one- or two-sided<br>*Only common tests should be described solely by name; describe more complex techniques in the Methods section.*
☐	☒	A description of all covariates tested
☐	☒	A description of any assumptions or corrections, such as tests of normality and adjustment for multiple comparisons
☐	☒	A full description of the statistical parameters including central tendency (e.g. means) or other basic estimates (e.g. regression coefficient) AND variation (e.g. standard deviation) or associated estimates of uncertainty (e.g. confidence intervals)
☐	☒	For null hypothesis testing, the test statistic (e.g. *F*, *t*, *r*) with confidence intervals, effect sizes, degrees of freedom and *P* value noted<br>*Give P values as exact values whenever suitable.*
☒	☐	For Bayesian analysis, information on the choice of priors and Markov chain Monte Carlo settings
☒	☐	For hierarchical and complex designs, identification of the appropriate level for tests and full reporting of outcomes
☐	☒	Estimates of effect sizes (e.g. Cohen's *d*, Pearson's *r*), indicating how they were calculated

Our web collection on statistics for biologists contains articles on many of the points above.

Software and code

Policy information about availability of computer code

Data collection	We used R package "RMySQL" (version 0.10.20) to obtain data from the dataset GloNAF and GIFT, and used R package "raster" (version 3.0-7) to get data from the database "Worldclim".
Data analysis	All the main data analysis were performed using R version 4.1.0. We used the R package "sf" (version 0.8-0) to unify polygons for GloNAF and GIFT and used the R package "geosphere" (version 1.5-10) to calculate geographic distance between regions. We used the R package "Taxonstand" (version 2.2) to standardise the taxonomic names to the Plant List. We transformed each bioclimatic variable to be as approximate to a normal distribution as possible using the R package "normalizer" (version 0.1.0). We used the R package "betapart" (version 1.5.1) to calculate Simpson and Sørensen dissimilarities between regional floras. We used the R package "spdep" (version 1.1-3). One pdf document including the R codes of the main analysis, and the relevant output of the codes is available at https://figshare.com/articles/dataset/Data_and_Code_for_Yang_et_al_The_global_loss_of_floristic_uniqueness/14991624.

For manuscripts utilizing custom algorithms or software that are central to the research but not yet described in published literature, software must be made available to editors and reviewers. We strongly encourage code deposition in a community repository (e.g. GitHub). See the Nature Research guidelines for submitting code & software for further information.

Data

Policy information about availability of data

All manuscripts must include a data availability statement. This statement should provide the following information, where applicable:

- Accession codes, unique identifiers, or web links for publicly available datasets
- A list of figures that have associated raw data
- A description of any restrictions on data availability

The core datasets of this study are available at https://figshare.com/articles/dataset/

Data_and_Code_for_Yang_et_al_The_global_loss_of_floristic_uniqueness/14991624. These include 1) similarity and degree of homogenization between pairwise regions, 2) geographic distance, climatic distance, and the administrative relationship between pairwise regions, 3) average degree of homogenization of each region, 4) characteristics of regions and their floras, including the richness and phylogenetic diversity of native and naturalized species, the proportion of endemic species, the donor score, the size of the region, and whether the region is an island or a mainland region, and 5) phylogeny of the seed plants with accepted names in TPL and a dataset describing the taxonomic group (e.g. family and order) of each species and how the species was added to the phylogeny if it was initially missing from the phylogeny. The GloNAF database has been published in a data paper6, and the most recent version is available upon request. Data from the GIFT database is available from co-authors PW and HK on request and the database will be made publicly available in due course. The original phylogeny ALLMB is available at https://github.com/FePhyFoFum/big_seed_plant_trees/releases. The dataset TRADHIST can be downloaded at http://www.cepii.fr/CEPII/en/bdd_modele/presentation.asp?id=32. The Plant List database can be accessed at http://www.theplantlist.org/1.1/browse/A/. The database Worldclim can be accessed at http://www.worldclim.com/version2. A detailed description of the databases and their access approaches can be found at Supplementary Table 4.

Field-specific reporting

Please select the one below that is the best fit for your research. If you are not sure, read the appropriate sections before making your selection.

☐ Life sciences ☐ Behavioural & social sciences ☒ Ecological, evolutionary & environmental sciences

For a reference copy of the document with all sections, see nature.com/documents/nr-reporting-summary-flat.pdf

Ecological, evolutionary & environmental sciences study design

All studies must disclose on these points even when the disclosure is negative.

Study description	By combining a global database of native plant inventories with one of naturalized alien plant inventories, we studied how the naturalization of plant species caused the taxonomic and phylogenetic homogenization in global floras. Our study includes nearly 200,000 plant species distributed in more than 650 regions around the world, which cover about two-thirds of the global ice-free land surface. The unprecedented high coverage of the data we assembled allowed us to quantify the global extent of the loss of floristic uniqueness by alien plants. We identified hotspots of floristic uniqueness loss and showed that these are linked with biogeographic drivers and human pressures.
Research sample	We used existing datasets. We extracted regional lists of alien species from the Global Naturalized Alien Flora (GloNAF) database (van Kleunen et al. 2019; Ecology; DOI: 10.1002/ecy.2542) and regional lists of native species from the Global Inventory of Floras and Traits (GIFT) database (Weigelt et al. 2019; Journal of Biogeography; doi: 10.1111/jbi.13623). The seed-plant phylogeny that we used is developed based on the phylogeny by Smith and Brown (2018; American Journal of Botany; doi: 10.1002/ajb2.1019). The explanatory variables are all from published datasets that are freely open to the public, for which we provided download links.
Sampling strategy	We used all data available for taxa with accepted names in The Plant List (http://www.theplantlist.org/). As this is not an experimental study and our study is at the global level, we used all available data from the global databases, there is no need to predetermine sample sizes.
Data collection	The GloNAF database and GIFT database were developed by the authors. Other datasets used in this study were downloaded by the corresponding author.
Timing and spatial scale	We downloaded the GloNAF and the GIFT databases on 23 September 2020 and downloaded the ALLMB phylogeny on 18 January 2019. We downloaded the Angiosperm taxa from the Plant List on 17 February 2020. We downloaded the database TRADHIST and the database Worldclim on 1 July 2019 and 14 March 2020, respectively. Each database was obtained by one-time downloading and finished within one day. All datasets downloaded and used in this study are at the global scale.
Data exclusions	To be able to align the different databases, all taxonomic names were standardized according to the names used in The Plant List with the R package "Taxonstand". The GloNAF and GIFT databases include regions with relatively incomplete native or naturalized taxa inventory. We excluded such regions to avoid overestimate or underestimate the homogenization between global regions by naturalized plant species.
Reproducibility	As we did not do an experiment, but analyzed existing databases, we did not test for reproducibility of experiments. However, we provided the key processed data and the R code that could be used to reproduce the main results of the study
Randomization	As we did not do an experiment, but analyzed existing databases, we did not have to allocate samples randomly to groups.
Blinding	As we did not do an experiment, but analyzed existing databases, blinding was not relevant to this study.

Did the study involve field work? ☐ Yes ☒ No

Reporting for specific materials, systems and methods

We require information from authors about some types of materials, experimental systems and methods used in many studies. Here, indicate whether each material, system or method listed is relevant to your study. If you are not sure if a list item applies to your research, read the appropriate section before selecting a response.

Materials & experimental systems

n/a	Involved in the study
☒	☐ Antibodies
☒	☐ Eukaryotic cell lines
☒	☐ Palaeontology and archaeology
☒	☐ Animals and other organisms
☒	☐ Human research participants
☒	☐ Clinical data
☒	☐ Dual use research of concern

Methods

n/a	Involved in the study
☒	☐ ChIP-seq
☒	☐ Flow cytometry
☒	☐ MRI-based neuroimaging
